# Dairy cows: in the age of the genotype, #phenotypeisking

**DOI:** 10.1093/af/vfaa004

**Published:** 2020-04-01

**Authors:** Mike Coffey

**Affiliations:** Animal and Veterinary Sciences, SRUC, Roslin Institute Building, Easter Bush, Midlothian, UK

**Keywords:** analytics, big data, dairy cattle, phenomics, sensors

**Implications**

- The limiting factor for future evaluations of new traits of economic importance is phenotypes.
- Genomics is an ideal technology used to spread the value of difficult-to-measure phenotypes throughout the population.
- There are many actors in the chain required to create that benefit and the creation of those value chains for new and difficult to measure traits is still in its infancy, but one thing is certain: Traditional mechanisms to pass evaluations back to nonrecording farmers will change in the future.

## Introduction

Estimated breeding values (**EBVs**) have historically been calculated by national evaluation centers from phenotypes available in large quantities from national recording organizations, usually if not exclusively made available for no charge. The usual deal is that there is no charge then made to phenotype contributors for them to use EBVs. Most countries have an extensive range of EBVs for traits of economic importance and many countries participate in Interbull whereby local evaluations are merged with all others internationally for the same bull and an evaluation produced on a local scale containing information for all bulls internationally even when that bull has no offspring in the local country.

Prior to the widespread deployment of genomic selection in dairy cattle, the priority for global breeding companies was to collect enough genotypes on older bulls to create an informative reference population. The reason for this was that the phenotype available to them was the Interbull proof or national proof for the bull for the range of traits recorded in that country. This meant that phenotypes were not a limiting factor and that genotypes were the limiting factor since they were expensive at the time and large numbers (~20,000) were needed for a reference population producing genomic breeding values (**gEBVs**) at sufficient accuracy. However, DNA is easily extracted from frozen semen and many stores of old semen existed. The availability of Interbull proofs from all over the world meant that any bull from any country could contribute to a national reference population, even if that bull had never been used in a particular country. This was how national reference populations have been created. However, once all old bulls have been genotyped that have phenotypes (progeny proofs or Interbull proofs), the limiting factor for future evaluations has become unavailable genotypes or phenotypes.

The rapid fall in price per genotype means that many young females and nearly all new (young) candidate bulls are routinely genotyped and genotypes are no longer limiting. It seems the limiting factor for future evaluations of new traits of economic importance is now phenotypes. You might paraphrase all that with “In the age of the genotype, #phenotypeisking.”

The benefits of genomic selection have been highlighted especially for those traits that are available only late in life (lifespan), are difficult (expensive) to measure (e.g., feed intake) or are not yet measurable in large volumes or at sufficient accuracy (e.g., methane emissions). These difficult to measure phenotypes are coincidentally for those traits that are of current global importance in respect of climate change and are required right now to enable farmers to make socially important selection decisions (i.e., cattle that produce food with less resource use and that have a lower environmental impact). Genomics is an ideal technology to be used to spread the value of those phenotypes throughout the population since young animals with no phenotypes but that have been genotyped can have a prediction for the important trait made from the reference population. A really good review of genomic selection and phenotypes is given by Cole and VanRaden (2018).

## Future Directions

So how will these difficult traits be measured? And by whom? Who will pay? And who will benefit?

Historically, genetic evaluations have been a by-product of national recording programs. The phenotypes collected for on-farm management were made available to national evaluation centers for processing into breeding values and were then passed back to farmers that recorded the phenotypes notionally for no charge for the breeding value (in essence a quid pro quo). Each country had a mechanism to pass the evaluations back to nonrecording farmers also which essentially meant every farmer had access to the breeding value for every trait. However, this model is set to change.

Take the example of feed intake in dairy cows. If we need feed intake for national evaluations, do we ask every farmer with milk records to install equipment and record feed intake? Impossible! Thus, we have to work out how to get enough phenotypes on informative animals that have been genotyped. This seems to suggest some form of nucleus. In some countries, this is organized by breeding companies (NLD, USA) and in other countries, it is an outcome of research facilities recording feed intake for research projects. The gDMI (global dry matter initiative) pooled records to produce an international dataset of genotypes from 6,000 cows with 10,000 phenotypes. This has created an international SNP key (set of equations based on the genotypes and phenotypes of the reference population) and genomic breeding values (gEBVs) for feed intake. The value of pooling records is described in Banos et al. (2012) and the benefits of so doing in de Haas et al. (2012). In Australia, the Dairy Information Nucleus (**DIN**) is an effort to gather feed intake (and other) records for genomic selection indices from contracted farms that genotype their cows (Pryce et al., 2018).

**Figure 1.**
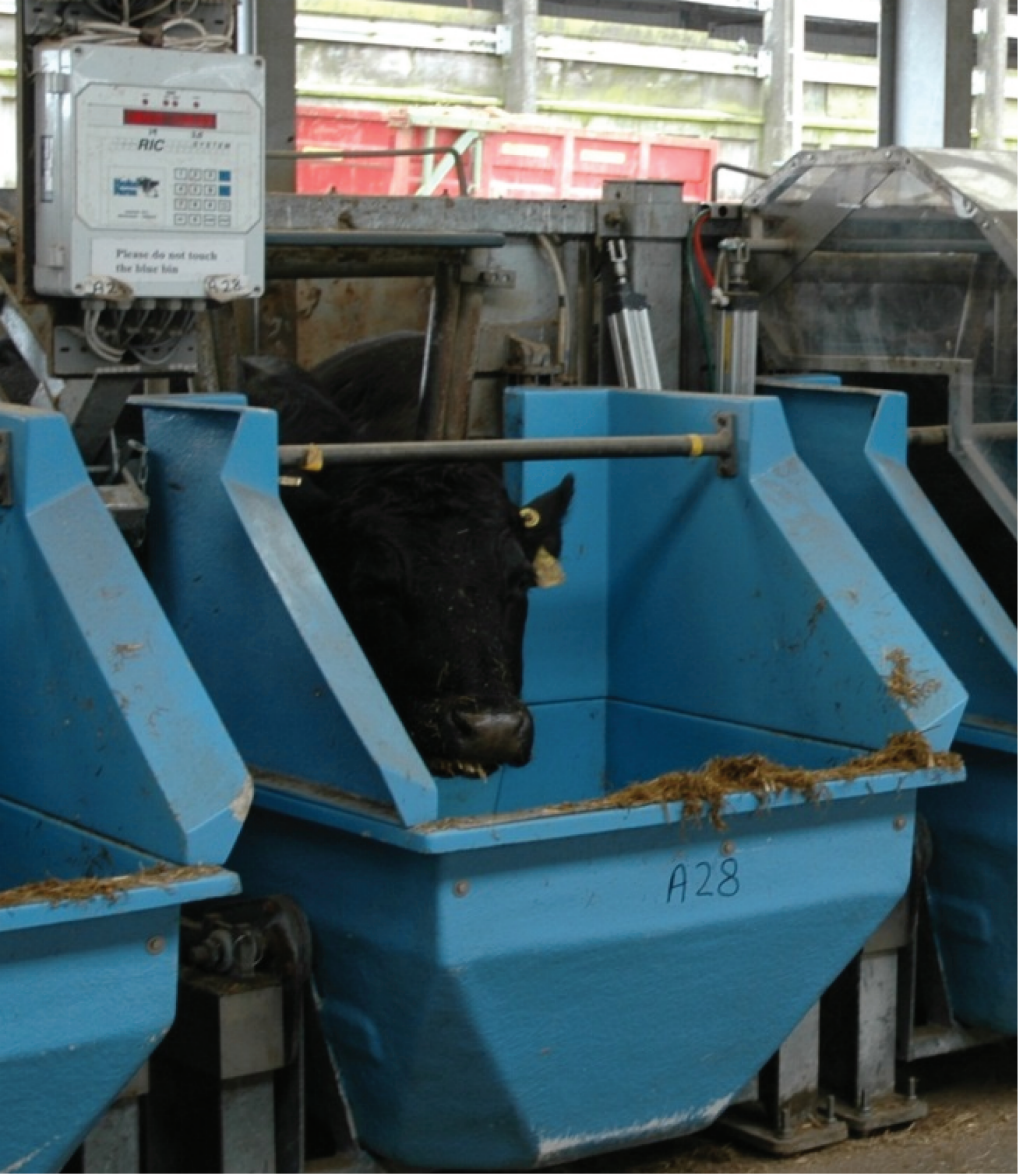
Equipment used to measure feed intake in individual cattle. This type of equipment has a gate that is activated as the cow approaches the equipment.

**Figure 2.**
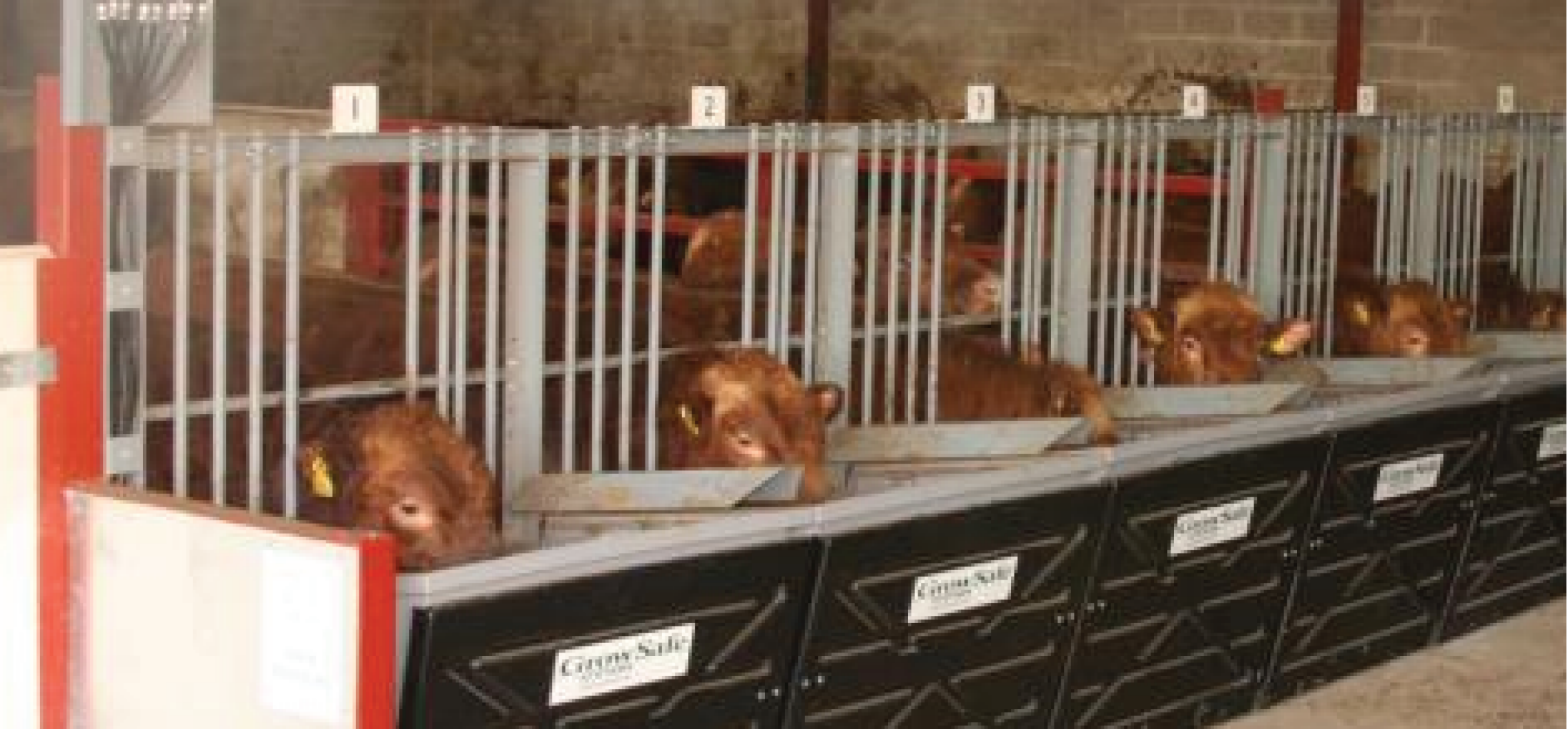
Example of equipment that measures feed intake in cattle. This type of equipment allows all cows to eat at each feed bunk.

**Figure 3.**
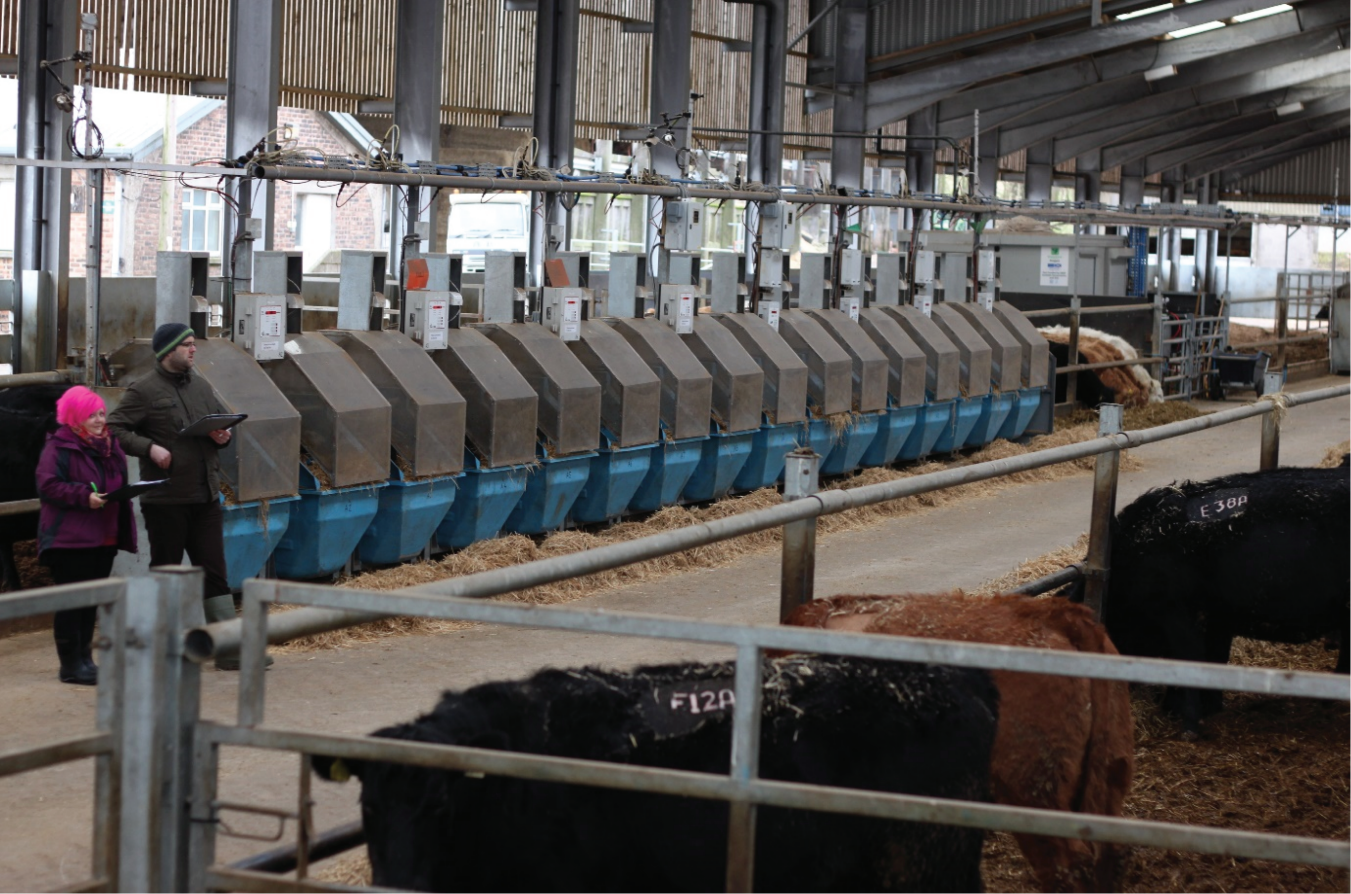
The equipment shown in this photo contains hoods to capture methane emissions from individual cattle.

Examples of feed intake recording equipment for cattle are shown in Figures 1–3. One type of equipment has a gate that is activated as the cow approaches allowing some cows to be restricted to some gates (Figure 1). Another type of equipment that measures feed intake allows all cows to eat at each gate (Figure 2). Figure 3 shows equipment with hoods added to allow capture of methane emissions from individual cows.

For future recording the questions arises as to what animals would be recorded? The value of each incremental feed intake record for a bull’s daughters declines once sufficient records have been obtained for a reasonably accurate EBV. Each new bulls’ first records have much higher value and each incremental record has less value. Gonzalez-Recio et al. (2014) attempted to calculate the value of incremental records which could be used as a framework to pay for future recording schemes. If the recording scheme is organized specifically for the purpose of creating phenotypes for genomic selection, those animals for recording will be identified as having the greatest information contribution to the existing dataset. If records are gathered for some other (research) purpose, then the information content may not be optimal for genomic evaluations but this information would be better than having no information.

### How will animals become part of the nucleus?

A nucleus can be a single large farm that genotypes all cows and records all available phenotypes of interest. In dairy farming, some single farms are sufficiently big to create their own nucleus. However, the general trend appears to be groups of farms being contracted to either a breeding company or a research project to become a distributed nucleus all recording to some predefined protocol allowing records to be merged and analyzed together. The DIN in Australia or the Langhill herd in United Kingdom are examples of farms that create large collections of phenotypes for genotyped cows. Some breeding companies have their own farms where data is recorded on daughters of their own bulls to be merged with data from other participating farms. It is easy to imagine a wide range of solutions that are driven by the circumstances prevalent in each country ranging from breeding company nuclei through to farmer coops.

### Who pays?

For breeding companies, the availability of information for breeding values cannot be left to chance. As such, many are seeking to secure datasets for breeding value estimation for traits that will provide a competitive advantage. An example is Immunity Plus marketed by Semex and Wellbeing from Zoetis. These indices will be by definition, proprietary, and will not be comparable across companies. In these situations, farmers will have to make decisions about which company they deal with on the basis of available breeding values. Some companies are now establishing their own network of phenotype providers. These are a mixture of their own farms that collect data specifically for them and contracted farms that have some commercial arrangement with them. These “phenotype farms” collect data that may have a value greater than the agricultural product they coincidentally produce.

In some countries, if farmers can be paid the value of the phenotype they collect this may provide an attractive route into farming for new entrants. Borrowing against a secure and predictable income stream such as sale of phenotypes under contract may enable greater borrowing potential and may increase the penetration of younger farmers into modern agriculture. Records for traits that are expensive to measure may be paid for by whomever contracts with the farmer. In some cases, it could be national evaluation centers; in other cases, it could be breeding companies. In countries that have well-integrated recording, evaluation, and breeding centers such as NLD and NZ, these issues are simply internal accounting procedures. In countries that are less integrated (e.g., United States, United Kingdom, AUS), there may be competition to procure phenotypes on important traits such as feed intake and disease traits but for other traits that are for notifiable diseases such as bovine tuberculosis; these phenotypes may not be available to commercial companies.

### Who benefits?

Clearly, everybody benefits from more efficient and healthy cows. The farmer will be the first to benefit but benefits spread through to consumer and society as a whole. This is especially true for efficiency-based traits and greenhouse gas (**GHG**) emissions. At present, no known system exists for rewarding farmers for reduced GHG emissions and so, farmers do not currently benefit directly. Once mechanisms are available to accurately record GHG emissions, it is anticipated that farmers will benefit for reducing GHG emissions from their dairy cattle.

For farmers to be able to breed more efficient cows that produce less GHGs, they need access to a SNP key at a price that makes selection beneficial. The SNP key therefore becomes the route by which the benefit is created for whole populations based on a small part of the population investing in it. The main difficulty will be in ensuring equitable distribution of benefits and costs across the supply chain. Will society get the benefits of more environmentally friendly dairy cows? Yes. Will they pay directly to farmers for that benefit? No. There are many actors in the chain required to create that benefit and the creation of those value chains for new and difficult to measure traits is still in its infancy.

## Conclusion

If I were a dairy or beef farmer, I would genotype all my animals and farm phenotypes for sale. Those phenotypes would be based around efficiency, resource use, disease, and product quality. #phenotypeisking

## About the Authors

**Mike Coffey** is a Professor of Livestock Informatics and Team Leader in the Animal Breeding and Genetics and EGENES Group at Scotland’s Rural Agricultural College. His main area of interest is dairy cattle breeding and identification of appropriate selection goals that meet as many stakeholder requirements as possible.

## References

[CIT0001] Banos G., Coffey M. P., Veerkamp R. F., Berry D. P., and Wall E.. 2012 Merging and characterising phenotypic data on conventional and rare traits from dairy cattle experimental resources in three countries. Animal 6:1040–1048. doi:10.1017/S1751731111002655. 23031463

[CIT0002] Cole J. B., and VanRaden P. M.. 2018 *Symposium review*: Possibilities in an age of genomics: the future of selection indices. J. Dairy Sci. 101:3686–3701. doi:10.3168/jds.2017-13335. 29103719

[CIT0003] Gonzalez-Recio O., Coffey M. P., and Pryce J. E.. 2014 On the value of the phenotypes in the genomic era. J. Dairy Sci. 97:7905–7915. doi:10.3168/jds.2014-8125. 25453600

[CIT0004] de Haas Y., Calus M. P., Veerkamp R. F., Wall E., Coffey M. P., Daetwyler H. D., Hayes B. J., and Pryce J. E.. 2012 Improved accuracy of genomic prediction for dry matter intake of dairy cattle from combined European and Australian data sets. J. Dairy Sci. 95:6103–6112. doi:10.3168/jds.2011-5280. 22863091

[CIT0005] Pryce J. E., Nguyen T. T. T., Axford M., Nieuwhof G., and Shaffer M.. 2018 *Symposium review*: Building a better cow-The Australian experience and future perspectives. J. Dairy Sci. 101:3702–3713. doi:10.3168/jds.2017-13377. 29454697

